# Trend in Hospital Admissions for Cardiovascular Diseases (CVDs) Before and During the Coronavirus Disease 2019 (COVID‐19) Pandemic: A Retrospective Analysis From a Sub‐Urban Area in Sub‐Saharan Africa

**DOI:** 10.1002/hsr2.71993

**Published:** 2026-03-08

**Authors:** Konfo Gaetan Kwasseu, Clovis Nkoke, B. F. Kuaguim Kenfack, Dzudie Anastase

**Affiliations:** ^1^ Faculty of Health Sciences University of Buea Buea Cameroon; ^2^ Clinical Research Education Networking and Consultancy Organization, CRENC Yaounde Cameroon; ^3^ University Teaching Hospital Angré Abidjan Ivory Coast; ^4^ Faculty of Medicine and Biomedical Science University of Yaounde I Yaounde Cameroon

**Keywords:** Burden, cardiovascular disease, coronavirus disease, mortality, outbreak

## Abstract

**Background and Aims:**

The COVID‐19 pandemic was a global public concern and constitutes a future threat to the world population due to its indirect effect on the burden of non‐communicable diseases. The pandemic manifested disruptions in healthcare delivery and access. However, there is limited data in Sub‐Saharan Africa on the impact of COVID‐19 on cardiovascular disease (CVD) admissions and outcomes. This study aimed to compare the trends of CVD admissions and outcomes before and during the COVID‐19 pandemic in the Southwest Region of Cameroon.

**Methods:**

We carried out a retrospective study of patients suffering from CVDs admitted from March 11, 2018, to March 11, 2020 (Pre‐COVID‐19 pandemic period) and from March 11, 2020, to March 11, 2022 (COVID‐19 pandemic period). A *p*‐value < 0.05 was considered statistically significant.

**Results:**

There were 483 admissions due to CVD during the COVID‐19 pandemic and 518 during the pre‐COVID‐19 period. There was no significant difference in mean age before (57.97 ± 15.6 years) and during the pandemic (59.74 ± 16.1 years) (*p* = 0.44). There was also no significant change in the proportion of males and females during and before the pandemic: males (21, 4%, and 24.8%), and females (26.8% and 27%), (*p* = 0.28). There was a downward secular trend with random variation in the number of CVD admissions during the pandemic compared with the corresponding pre‐COVID period, which had an upward trend. Rates of admissions of Acute Myocardial Infarction decreased the most (22.2%) during the first wave of the pandemic. The in‐hospital mortality increased by 2.4% with a relative risk for Mortality of 1.18 (95% CI [0.87–1.61], *p* = 0.28). There was no change in median length of hospital stay (*p* = 0.936).

**Conclusion:**

This study provides evidence of a decreasing tendency in admissions due to CVD during the COVID‐19 Pandemic at the BRH. The effects varied among the different types of CVDs. The in‐hospital mortality of CVDs did not change significantly.

**Abbreviations**
COVID‐19: coronavirus disease 2019
CVD: cardiovascular diseases
NCD: noncommunicable diseases

## 1 Background

Non‐communicable diseases (NCDs) contribute about 70% of deaths worldwide, with approximately 80% of these occurring in low‐ and middle‐income countries [1]. Among NCDs, cardiovascular diseases (CVDs) are a leading cause of death and disability globally, accounting for more cases each year than all other causes combined [2]. Due to their chronic and often life‐long nature, CVDs typically require repeated interactions with health systems; lacking access to timely care frequently results in devastating consequences for those affected [3].

SARS‐CoV‐2 primarily targets the respiratory system but also negatively impacts the cardiovascular system [4]. Consequently, COVID‐19 patients often exhibit various cardiovascular‐related clinical features, including myocardial injury, myocarditis, acute coronary syndrome (ACS), acute myocardial infarction (AMI), arrhythmias, cardiac arrest, venous thromboembolism, and heart failure [5]. SARS‐CoV infections are known to reduce ACE2 expression, which may lead to myocardial dysfunction and broadly impact the cardiovascular system [6]. A 12‐year longitudinal study of 25 individuals who recovered from SARS‐CoV infection found that 68% developed hyperlipidemia, 44% experienced cardiovascular disorders, and 60% showed abnormalities in glucose metabolism [7, 8]. Also, Previous research on SARS‐CoV has shown that patients with pre‐existing conditions like heart disease and diabetes had a higher risk of mortality [9]. Data from the National Health Commission of China (NHC) indicate that among individuals who died from COVID‐19, 17% had a history of coronary heart disease and 35% had hypertension [8]. CVD is more common in older individuals, and the associated decline in immune system function may further increase the risk of severe COVID‐19 outcomes [10].

Several studies have suggested a link between CVD and severe COVID‐19, but limitations in national surveillance and standardized data hinder precise estimates [11]. A systematic analysis of six studies in China found elevated rates of hypertension (17.1%), cardio‐cerebrovascular disease (16.4%), and diabetes (9.7%) among COVID‐19 patients, with cardio‐cerebrovascular disease notably higher than in the general population [12]. Additionally, evidence shows that cardiovascular complications and elevated cardiac biomarkers are common in severe COVID‐19 cases, indicating significant cardiac involvement during disease progression [13, 14].

Since the declaration of the COVID‐19 pandemic, global trends in NCD admissions have changed significantly [7]. Beyond its direct health impact, the pandemic has indirectly influenced morbidity and mortality by altering patient and provider behaviors and through the reorganization of health systems [15]. In particular, patients with NCDs such as CVDs have faced interruptions in treatment, increasing the risk of complications due to poor chronic disease management. Studies from high‐income countries have shown reduced cardiovascular consultations and admissions during the pandemic [15, 16, 17].

In low‐ and middle‐income countries (LMICs) such as Cameroon, these challenges are likely magnified due to resource‐limited health systems and longstanding healthcare inequities. However, there is a striking lack of data examining this issue in Africa generally, and Cameroon specifically. The few available studies suggest reductions in the delivery of cardiology services and widespread adaptation of services to the constraints of the pandemic [18, 19]. Many patients have avoided seeking care for CVDs due to fear of contracting COVID‐19 in healthcare facilities. Since the first confirmed case in Cameroon, the country has experienced rising case numbers and high mortality [20], fueling public fear and leading many to desert hospitals [19].

Given the already high and unmet burden of CVD in Cameroon, and the global concern regarding rebounds in cardiovascular admissions and mortality, it is crucial to examine the pandemic's indirect effects on CVD burden and health service delivery [15, 18, 19].

To our knowledge, this is the first study in the Southwest region of Cameroon comparing trends in CVD admissions before and during the COVID‐19 pandemic. Our findings have the potential to fill a critical gap in evidence from LMICs and inform policymakers and healthcare providers about the pandemic's broader consequences on CVD care. By quantifying these trends, this work can guide strategies to mitigate the impact of future health emergencies on chronic disease care and improve preparedness in similar contexts. This novel focuses on pandemic‐driven shifts in CVD admissions in a region where such data is sparse, highlighting the urgent need to address disparities in health service delivery and plan for resilient healthcare systems.

## 2 Methodology

### 2.1 Study Setting and Design

We conducted a retrospective study on patients admitted at the Internal Medicine Department of the Buea Regional Hospital (BRH) from March 11, 2018, to March 11, 2020 (Pre COVID‐19 Pandemic), and from March 11, 2020, to March 11, 2022 (During COVID‐19 Pandemic). The BRH is a secondary‐level hospital that serves as the main referral center for patients suffering from CVDs in the Southwest region of Cameroon. It has a capacity of about 111 beds and a catchment population of about 200,000 inhabitants. The Internal Medicine Department has a consultant cardiologist, two internists, and general practitioners who manage CVDs using appropriate and standard methods. This facility also serves as a diagnostic and treatment center for patients suffering from COVID‐19 infection. Buea is the headquarters of the Southwest region of Cameroon. It is a semi‐urban setting with the main economic activity as agriculture [20].

### 2.2 Data Collection

We collected data on patients admitted for CVDs at the BRH 2 years before and after the declaration of COVID‐19 as a pandemic in Cameroon. The data collection form was designed using Epi Info version 7.2.5.0 and was used to extract data from the hospital records of affected patients. After identifying the records of each patient suffering from CVD, the diagnosis on entry was identified. The progress in the ward, the investigations made, and the final diagnosis of CVD made by the specialist on discharge were noted. We included records of patients with the diagnosis of the CVD of interest. We excluded those with no clear diagnosis of CVD in the file. The following data were collected: Sociodemographic data: age, gender, occupation, and comorbidities; Clinical data: final diagnosis of the patient (type of cardiovascular disease and subtype); Outcome: outcome of the patients (death, full recovery, sequelae, or partial recovery discharge), and duration of hospitalization. We considered the following CVDs: stroke, heart failure, acute myocardial infarction, uncontrolled hypertension, pericardial disease, arrhythmias, and venous thromboembolism. Patient information was coded to ensure confidentiality, and the collected data was stored in a password‐protected computer.

### 2.3 Sample Size and Statistical Analysis

A convenient sampling of all eligible participants was considered for this study. The data were analyzed using SPSS version 25. Continuous variables were presented as means with standard deviations, while categorical variables were summarized using frequencies and percentages. The normality of continuous variables was assessed using the Shapiro‐Wilk test and visual inspection of histograms and Q–Q plots. For normally distributed variables, comparisons between two groups were performed using the Student's *t*‐test. The Mann–Whitney *U* test was used for non‐normally distributed continuous variables, such as the median length of hospital stay before and during the COVID‐19 period.

The chi‐square test was used to assess differences in the frequency of CVD admissions between the pre‐COVID‐19 and COVID‐19 periods, as well as between the first and second waves of the pandemic in Cameroon. These results were presented in tabular form. The relative risk for mortality during the pandemic compared to the pre‐pandemic period was calculated using the observed mortality rates in both groups. A 2 × 2 contingency table was used to derive the relative risk, 95% confidence interval, and corresponding *p*‐value and the difference in mortality rates between the two study periods was also evaluated using the chi‐square test. A *p*‐value of < 0.05 was considered statistically significant. All reported p‐values were based on two‐sided hypotheses.

### 2.4 Ethical Considerations

Ethical clearance for this study was obtained from the Institutional Review Board (IRB) of the Faculty of Health Sciences, University of Buea (Approval No. 1607‐01), dated 2nd February 2022. Administrative authorization was also secured from the Southwest Regional Delegation of Public Health and the administration of Buea Regional Hospital.

## 3 Results

**Figure 1:**
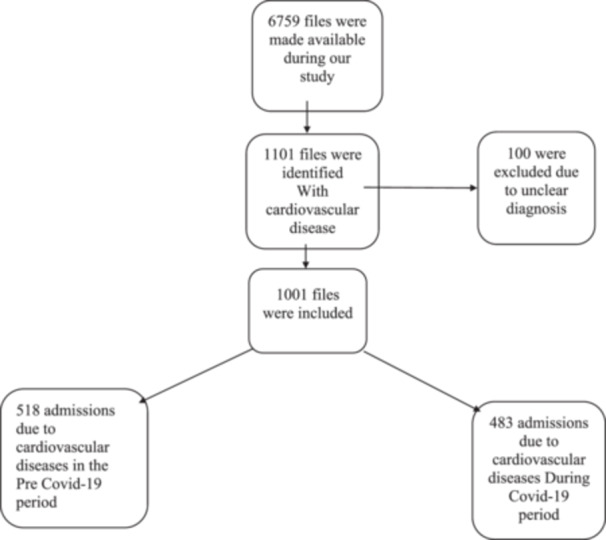
Study flow‐chart.

**Figure 2:**
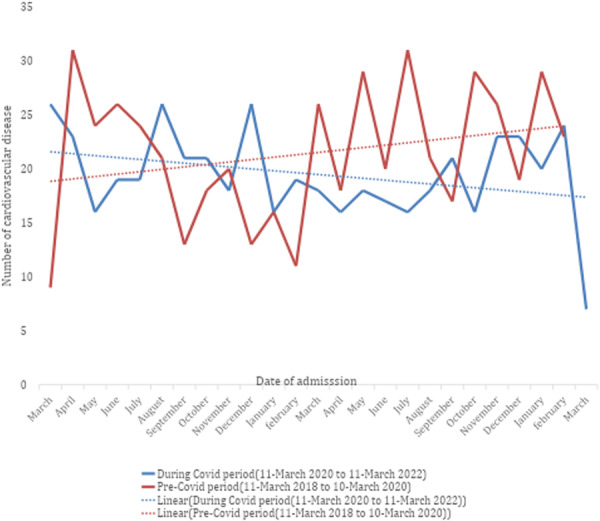
Comparing trends in cardiovascular disease admissions before and during the COVID‐19 pandemic.

Figure 1, shows the flow diagram of study participants, out of the 6759 files made available, 1001 were eligible for this study. The mean age of patients admitted with a CVD was 57.97 ± 15.60 years during the pre‐COVID‐19 pandemic period compared to 59.74 ± 16.10 during the COVID‐19 pandemic period (*p* = 0.442). The number of males during the pandemic was 215 (21.5%) compared to 248 (24.8%) before, while that of females was 268 (26.8%) during the pandemic compared to 270 (27%) before, giving a male‐to‐female ratio during the COVID‐19 pandemic of 0.9 compared to 0.8 during the pre‐pandemic period (Table 1). As shown in figure 2, there was a downward secular trend with random variation in the number of CVD admissions during the COVID‐19 period starting from March 2020, as compared with the corresponding pre‐COVID period, which showed an upward trend. Figure 2 also shows that admissions declined sharply from April to June 2020 and from April to August 2021. Overall, the rate of admissions of stroke decreased by 5.4%, Uncontrolled hypertension by 27.4%, pericarditis by 66%, and arrhythmias by 28.2%, while Heart failure admissions increased by 0.6% and acute MI by 3.4%. (Table 2). During the first wave of the COVID‐19 pandemic from March to September 2020, there was a 3% decrease in the proportion of strokes, a 7.2% decrease in the proportion of uncontrolled hypertension, a 22.2% decrease in acute MI admissions, and a 4.4% decrease in admissions due to venous thromboembolism compared to the corresponding period before the pandemic. There was also an increase in the proportion of heart failure by 3.5% with 5.2% and 14.3% respective increases in admissions due to arrhythmias, and pericardial disease when compared to the corresponding period pre‐COVID period (Table 3). The in‐hospital mortality rate for patients suffering from CVDs was 15.3% during the pandemic and 12.9% before the COVID‐19 pandemic (*p* = 0.446). The relative risk for Mortality during the Pandemic compared to the Pre‐pandemic was 1.18 (95% CI [0.87, 1.61], *p* = 0.280. However, this difference did not meet the conventional levels of statistical significance. Patients suffering from heart failure and stroke had the highest mortalities before and during the pandemic (Table 5). There was no difference in the median length of hospital stay before and during the COVID‐19 pandemic [7 (3–10) days vs 7 (4–10) days, (*p* = 0.936)) (Table 4).

**Table 1:** Sociodemographic characteristics of the study population.

		*N* (total)	Pre COVID‐19 period (march 11, 2018‐march 11, 2020) (%)	During COVID‐19 period (march 11, 2020‐march 11, 2022) (%)	*p*‐value
Age in years, mean (SD)		58.37 (15.882)	58.74 (16.118)	57.97 (15.633)	0.442
Sex	Male	463 (46.3)	248 (24.8%)	215 (21.5%)	0.286
	Female	538 (53.7)	270 (27.0%)	268 (26.8%)	

**Table 2:** Proportions of CVDs before and during the Covid‐19 pandemic.

Variables	*N* (total)	Pre‐COVID‐19 period (march 11, 2018‐march 11, 2020) (%)	During COVID‐19 period (march 11, 2020‐march 11, 2022) (%)	*p*‐value
Cardiovascular disease				
Stroke	338 (31.6%)	160 (47.3)	178 (52.7)	**0.046**
Heart failure	346 (32.3%)	174 (50.3)	172 (49.7)	0.502
Uncontrolled hypertension	234 (21.9%)	85 (36.3)	149 (63.7)	**< 0.001**
Acute MI	29 (2.7%)	15 (51.7)	14 (48.3)	0.998
Pericardial diseases	12 (1.1%)	2 (16.7)	10 (83.3)	**0.014**
Venous thromboembolism	34 (3.2%)	17 (50)	17 (50)	0.836
Arrhythmias	64 (6.0%)	23 (35.9)	41 (64.1)	**0.009**

Abbreviation: MI, myocardial infarction.

**Table 3:** Proportions of different cardiovascular diseases.

Cardiovascular disease	1st wave COVID‐19 *n* = 159 Frequency (%)	Corresponding period *n* = 168 Frequency (%)	*p*‐value
Stroke	45 (17.2)	53 (20.2)	0.052
Heart failure	64 (22.5)	54 (19)	**0.004**
Uncontrolled hypertension	32 (20.9)	43 (28.1)	0.175
Acute MI	1 (5.6)	5 (27.8)	0.607
Pericardial disease	3 (21.4)	1 (7.1)	0.521
Venous thromboembolism	3 (13)	4 (17.4)	0.648
Arrhythmias	11 (19)	8 (13.8)	0.396

*Note: n* = total number of admissions, 1st wave of COVID‐19 in Cameroon: March 2020 to September 2020.

Abbreviation: MI, myocardial infarction.

**Table 4:** Outcome of cardiovascular diseases.

Outcome, *n* (%)	Total *n* = 1001 Frequency (%)	Pre‐COVID 19 period *n* = 518 Frequency (%)	During COVID‐19 period *n* = 483 Frequency (%)	*p*‐value
Alive/Discharged	857 (85.6)	450 (86.9)	407 (84.3)	0.446
Death	141 (14.1)	67 (12.9)	74 (15.3)	
Transfer to other facilities	3 (0.3)	1 (0.2)	2 (0.4)	
Median length of hospital stay in days	7.00	7.00	7.00	0.936
Interquartile range of length of hospital stay in days	4–10	3–10	4–10	

*Note:* Total number of admissions.

**Table 5:** Mortality due to different CVDs.

Cardiovascular disease	In‐hospital mortality before COVID *N* (%)	In‐hospital mortality during COVID‐19 (%)	*p*‐value
Stroke	25 (15.6)	28 (15.7)	0.222
Heart failure	28 (16.1)	31 (18.0)	0.128
Uncontrolled hypertension	9 (6.0)	4 (4.7)	**0.003**
Acute MI	1 (6.7)	3 (21.4)	0.463
Pericardial disease	1 (50.0)	3 (30.0)	0.118
Venous thromboembolism	4 (23.5)	4 (23.5)	0.186
Arrhythmias	4 (17.4)	7 (17.1)	0.515

Abbreviation: MI, myocardial infarction.

## 4 Discussion

This study examined the impact of the COVID‐19 pandemic on hospital admissions and outcomes of patients with cardiovascular diseases (CVDs) in a suburban internal medicine service in Cameroon. Three major findings emerged:

1. No significant difference was observed in the mean age and sex distribution of patients admitted before and during the pandemic.
2. A marked decline in CVD admissions was noted during the pandemic, particularly from April to June 2020 and April to August 2021.
3. In‐hospital mortality among CVD patients increased during the pandemic, despite no significant change in the median length of hospital stay.

Regarding patient demographics, our findings show that neither the mean age of patients nor the male‐to‐female ratio changed significantly between the two periods. This indicates that the pandemic did not alter the demographic profile of patients presenting with CVDs. Similar findings have been reported in other regional studies, such as those conducted in Brazil and South Africa, suggesting that demographic characteristics remained stable even as healthcare‐seeking behavior and outcomes changed [21].

Furthermore, we observed a notable decline in CVD admissions during the pandemic. The most pronounced drop occurred during the initial lockdown phase and during the second wave, likely reflecting the public's fear of contracting COVID‐19 in hospitals, as well as structural barriers such as lockdown measures and transportation restrictions [22]. Cultural factors, including the fear of improper burial and lack of final rites, played a major role in many African communities and may also have contributed to patients avoiding hospital visits [16]. This is because mourning for the deceased is very significant in Africa, and the method is different than in Western countries [19, 23]. This timeframe also coincided with the implementation of 19 measures by the government during the lockdown to minimize the spread of the virus, including restrictions on access to public spaces [24]. Furthermore, with the introduction of the COVID‐19 vaccination, people were terrified and apprehensive about having the vaccine and hence sought to avoid visiting hospitals. This could have led to a decrease in the number of persons seeking healthcare during this era. Healthcare reconfiguration of both primary and secondary care by sudden discontinuation of routine face‐to‐face encounters, suspension of established care pathways, and reduced availability of medical staff has also contributed to the drop in cardiovascular disease consultations [25].

Similar declines in cardiovascular admissions have been reported globally. Normando et al. documented a 15% decrease in CVD admissions in Brazil [26], while Nganou et al. reported reduced cardiovascular consultations among African cardiologists during the same period [18]. Reductions in pediatric hospital visits during the pandemic in Cameroon further support the broader impact of COVID‐19 on hospital utilization [19]. The decrease in the proportion of stroke (3%), uncontrolled hypertension (7.2%), MI (22.2%), and venous thromboembolism (4.4%) during the first wave of the pandemic could be attributed to the fact that this was the early phase of the pandemic, little was known about the disease, and the population's fear of contracting the disease was at its peak during this period [23, 27], This finding is consistent with that of Akhtar N et al. in Qatar, who found that during the early phase of the COVID‐19 epidemic, stroke admissions decreased from 87 to 34 per month [28].

Healthcare system reorganization, including reduced outpatient services, suspension of routine consultations, and staff shortages, likely exacerbated this drop. In addition, misinformation, vaccine hesitancy, and fear of side effects may have discouraged individuals from seeking care, even when experiencing cardiovascular symptoms.

However, The increase in the proportion of heart failure 3.5%, arrhythmias 5.2%, and pericardial disease 14.3% during the first wave may be due to the fact that this period coincides with the lockdown period, which has been associated with increased risk of cardiovascular disease by promoting increases in unhealthy eating habits, decreases in physical activity, and stress [29, 30, 31, 32]. During the COVID‐19 lockdown, tobacco use increased in Italy, India, South Africa, the United Kingdom, and the United States [33]. However, this finding contradicts that of Hall ME et al, who reported a significant drop in admissions for heart failure from March to April 2020 [34, 35].

Lastly, we also found that in‐hospital mortality among CVD patients increased during the pandemic, although the length of hospital stay remained unchanged. This could be attributed to delayed hospital presentation, with patients arriving in more advanced stages of illness [23, 26]. Delay in seeking medical care during this period was observed in many hospitals around the world, including in wealthy countries [36]. Multiple studies have shown similar patterns: De Rosa et al. in Italy reported increased fatality rates for acute myocardial infarction during the pandemic [37], and Chelo et al. in Cameroon observed a doubling of pediatric mortality rates during the COVID‐19 period [19]. Delayed care may have been influenced by financial barriers, reduced mobility, or reliance on alternative therapies, all of which are well‐documented in both low‐ and high‐income countries [29, 38, 39].

Beyond healthcare access issues, biological mechanisms associated with COVID‐19 infection likely contributed to poorer cardiovascular outcomes. Conditions such as diabetes, hypertension, and obesity, which are common comorbidities among COVID‐19 patients, have been associated with higher mortality rates [40]. Furthermore, endothelial dysfunction, heightened inflammation, and hypercoagulability have been implicated in worsening clinical outcomes [40]. Viral infections, including SARS‐CoV‐2, can provoke arrhythmias, myocarditis, and other complications through direct myocardial injury and cytokine storm syndromes [41, 42, 43, 44]. Recent studies also suggest that immunological mechanisms contribute significantly to COVID‐19–related cardiovascular complications. Excessive inflammatory responses and cytokine activity induced by SARS‐CoV‐2 can promote myocardial injury, endothelial dysfunction, and coagulation disturbances, all of which may worsen cardiovascular outcomes. A range of immune‐inflammatory biomarkers has been investigated in COVID‐19 patients to support the monitoring and management of those with cardiovascular disease. These include complete blood counts, d‐dimer, APTT, prothrombin time, cytokine profiles, and inflammatory markers such as CRP, fibrinogen, ferritin, IL‐2, IL‐6, IL‐7, and TNF‐α [45, 46, 47]. These immunological interactions highlight the importance of considering inflammatory pathways when interpreting CVD‐related findings in COVID‐19 patients [14].

Moreover, another study reported a dysregulation of circulating monocytes and natural killer (NK) cells during COVID‐19 that persisted after recovery, as well as a correlation with plasma oxidized low‐density lipoprotein (ox‐LDL) levels [48]. These findings suggest that COVID‐19–related NK cell activation may influence lipid uptake, potentially contributing to increased foam cell formation and, consequently, elevated cardiovascular (CV) risk [48].

Several studies have described a general lymphocyte depletion during acute COVID‐19, predominantly affecting the T‐cell compartment, particularly in severe cases [49]. A decrease in naïve CD8+ T cells has also been reported by other authors, especially during the acute phase of the disease [50, 51]. However, these studies were largely limited to the acute phase. In contrast, Rajamanickam et al. evaluated the longitudinal course of lymphocyte subsets up to 6 months after primary SARS‐CoV‐2 infection and reported a persistent trend toward lower counts of naïve T cells (both CD4+ and CD8+) during convalescence [52].

Consistently, several investigations have documented persistent alterations in lymphocyte profiles among convalescent COVID‐19 patients, reflecting a shift toward a more senescent and/or exhausted immune phenotype [53, 54, 55]. Such changes may impair the availability and regenerative capacity of naïve T cells, particularly in individuals recovering from moderate‐to‐severe infection or experiencing long COVID. Additionally, advancing age has been associated with a decline in naïve lymphocytes, especially T cells [56]. Collectively, these mechanisms may contribute to an increased risk of cardiovascular complications.

One of the strengths of our study is the comparative design between the pre‐pandemic and pandemic periods, including detailed analysis of the first COVID‐19 waves and its corresponding pre‐pandemic period. The use of hospital registry data ensures consistency in record‐keeping and allows a reliable assessment of trends over time. However, some limitations must be acknowledged. As a single‐center study, the generalizability of our findings is limited, and they may not represent nationwide patterns. Patients with severe cardiovascular events who died at home were not captured in hospital records, potentially underestimating the true burden of disease. Variables such as medication adherence, socioeconomic status, and health‐seeking behavior, all of which may have influenced outcomes, were not assessed. Additionally, our dataset does not include the total population at risk, so incidence rates or incidence rate ratios could not be calculated; instead, we report absolute counts and relative changes as a cross‐sectional comparison.

Despite these limitations, this study provides valuable insights into the epidemiological impact of COVID‐19 on cardiovascular disease management in a lower‐middle‐income country. Our findings emphasize the need for resilient health systems and proactive strategies to maintain essential care services during public health emergencies.

## 5 Conclusion

This study suggests a decrease in cardiovascular disease admissions during the COVID‐19 pandemic at BRH. These findings may reflect reductions in healthcare utilization, possibly due to patient fear of infection in medical facilities. However, the results should be interpreted cautiously, given the study's observational design and limited sample size. Nevertheless, they highlight the importance of public health initiatives to encourage timely care seeking and the need for health system preparedness for future pandemics.

## Author Contributions

Dzudie Anastase conceptualized and supervised the research project, Clovis Nkoke contributed to data analysis and interpretation of results and writing of the manuscript, B.F. Kuaguim Kenfack contributed to the writing of the manuscript as well as data analysis, K. Gaetan Kwasseu data collection, analysis, and writing of the manuscript.

## Funding

The authors received no specific funding for this work.

## Disclosure

All authors have read and approved the final version of the manuscript, Kwasseu konfo gaetan had full access to all the data in this study and takes complete responsibility for the integrity of the data and accuracy of the data analysis.

## Ethics Statement

Ethical clearance for this study was obtained from the Institutional Review Board (IRB) of the Faculty of Health Sciences, University of Buea, under approval number 1607‐01, dated 2nd February 2022. Administrative authorization was also obtained from the Southwest Regional Delegation of Public Health and the administration of Buea Regional Hospital. The IRB waived the requirement for individual informed consent because the study was based on a retrospective review of anonymized hospital records, with no direct patient contact or intervention. All data were handled with strict confidentiality, and no personally identifiable information was collected or reported.

## Consent for Publication

The need for consent to participate was waived by the institutional review board of the faculty of health Sciences University of Buea.

## Conflicts of Interest

The authors declare no conflicts of interest.

## Transparency Statement

The lead author B. F. Kuaguim Kenfack affirms that this manuscript is an honest, accurate, and transparent account of the study being reported; that no important aspects of the study have been omitted; and that any discrepancies from the study as planned (and, if relevant, registered) have been explained.

## Data Availability

The datasets used and/or analyzed during the current study are available from the corresponding author upon reasonable request.
